# A cross-sectional study on obesity and related risk factors among women of the central market of Lusonga in Lubumbashi, Democratic Republic of Congo

**DOI:** 10.11604/pamj.2017.28.157.13762

**Published:** 2017-10-19

**Authors:** Paul Mawaw, Thierry Yav, Olivier Lukanka, Olivier Mukuku, Christian Kakisingi, Jean-Baptiste Kakoma, Oscar Numbi Luboya

**Affiliations:** 1 Department of Public Health, University of Lubumbashi, DRC; 2 Department of Research, High School for Medical Technics of Lubumbashi, DRC; 3 Department of Internal Medicine, University of Lubumbashi, DRC; 4 Department of Gynecology and Obstetrics, University of Lubumbashi, DRC

**Keywords:** Obesity, risk factors, women, Lubumbashi

## Abstract

**Introduction:**

Obesity is known as one of adjuvant factors for increase in non-communiable diseases (NCDs). The aim of this study was to describe the prevalence of obesity and identify its risk factors among women of the central market of Lusonga in Lubumbashi, Democratic Republic of Congo.

**Methods:**

In October 2014, we interviewed a total of 430 women selling in the central market of Lusonga in Lubumbashi. Data on sociodemographic characteristics, health-related habits and behaviors, diet, physical activity, chronic diseases, blood pressure and anthropometric measurements were collected. A multivariate logistic regression model was fitted.

**Results:**

Prevalence of overweight and obesity was 16.51% and 13.26% respectively. The logistic regression did not show any significant association between age and obesity. Risk of obesity was lower in married women (adjusted odds ratio (aOR) = 0.23 (0.08-0.63)). Women with low educational profile (primary school or less) were more likely to be obese than those with higher education (secondary or high school) (aOR = 2.50 (1.12-5.63)). Risk of obesity increased with living in urban area (aOR = 2.52 (1.00-6.36)), use of oral birth control pills (aOR = 11.07 (3.52-34.83)) and low consumption of fruit (aOR = 5.47 (1.88-15.92)) and vegetable (aOR = 2.42 (1.05-5.56)). Obese women were more likely to be hypertensive than non-obese (aOR = 7.15 (2.46-20.75)) and diabetics (aOR = 3.62 (1.62-8.11)).

**Conclusion:**

This study has reported a prevalence of 13.26% of obesity among women selling at Lusonga's market. Marital status, education level, residence, use of oral birth control pills and consumption of fruit and vegetables had a significant association with the prevalence of obesity in this category of women.

## Introduction

Non-communicable diseases (NCDs) are now major health and development challenges of the 21^st^ century, in terms of both human suffering and harm they inflict on the socio-economic fabric of vulnerable countries, particularly low and middle-income countries (LMICs) [1]. They were responsible for an estimated 38 million (68%) of the world's 56 million deaths in 2012 [2]. According to World Health Organization (WHO), the number of these deaths will increase from 38 million to 52 million annually by 2030 [3]. Main NCDs attributable to common risk factors are: cardiovascular diseases, cancer, chronic respiratory diseases and diabetes mellitus. These 4 NCDs alone accounted for 60% of all deaths and 47% of the global burden of the disease in 2005 [4] and together, they represent 82% of all NCDs related deaths [1]. One of adjuvant factors for increase in NCDs is the growing prevalence of overweight and frank obesity that have joined malnutrition and infectious diseases as major health threats for the developing world in the last decade [5]. Obesity increases the likelihood of diabetes, hypertension, cardiovascular diseases, stroke, cancers, obstructive sleep apnea and osteoarthritis. Overweight and obesity i.e. body mass index (BMI) ≥ 25kg/m² and ≥ 30kg/m² respectively-were estimated to account for 3.4 million deaths and 93.6 Disability Adjusted Life Years (DALYs) per year in 2010 [6]. The risk of comorbidities in adult population increases with a BMI in the range 25.0-29.9 kg/m² and the risk is moderate to severe with a BMI greater than 30 kg/m² [7]. In 2014, 11% of men and 15% of women worldwide were obese and in all WHO regions, women are more likely to be obese than men. In the African, South-East Asia and Eastern Mediterranean regions, the prevalence of obesity in women is roughly double than in men [1]. As per the WHO, the Democratic Republic of Congo (DRC) ranks among countries where the prevalence of obesity in people aged 18 years and more is considered to be less than 5% in men and 5 to 14.9% in women [1]. However, very little data on the burden of overweight and obesity in the DRC are available.

A survey on NCDs risk factors in Kinshasa showed a prevalence of 18.3% for overweight and 5.7% for obesity in both sex [8]. A baseline health survey conducted in 2008 in Fungurume (South of DRC) revealed a prevalence of 12.7% and 4.7% for overweight and obesity, respectively, in both sexes [9]. In many developing societies, the adoption of a Western lifestyle, with decreased physical activity and high caloric intake, is contributing to an alarming epidemiological transition marked by the shift in the leading causes of death from communicable, to NCDs [10, 11]. The recognition of the role of elevated BMI in these changes has made obesity a high priority for health authorities in the world [12]. Globalization of food market, urbanization and economic growth are the main drivers of this development [13]. In developing countries, deep societal changes and new behavioral patterns have emerged during the last decades and affect nutritional patterns [14]. The typical trend in the LMICs as per the nutrition transition is characterized by increased consumption of cheap vegetable oil, sugar, fatty meat, milk, processed food and soft drinks together with an increase in the consumption of food away from home [15]. At the same time, changes toward a sedentary lifestyle and less physical activity take place. Thus, not only diet, but the whole environment leads to obesity [16]. Women who sales in markets usually come from low-income settings and they spend all day sitting. They are more likely to be exposed to consumption of processed food, share of food away from home, and physical inactivity. In DRC as well as in many African countries, the social perception of body shape and size can be decisive to behavior for women and overweight has been associated with wealth, health, beauty and a positive connotation [17, 18]. The aim of this study was to describe the prevalence of obesity and its association with age, literacy, marital status, history of heart disease and diabetes mellitus, smoking, alcohol intake, fruit and vegetables intake, physical activity and use of oral birth control pills, among women selling in the central market of Lusonga in Lubumbashi, DRC.

## Methods

**Study design, population and sampling**: A descriptive cross-sectional study was conducted on a sample of 430 women both living in Lubumbashi and selling in the central market of Lusonga. Size of the sample was obtained with the sample size calculator recommended by WHO for most NCDs STEP studies [19]. We have used an average prevalence of 50%-as none or very little information on the prevalence of obesity are available for Lubumbashi-a margin of error of 0.05; a confidence interval of 95% and a design effect of 1 as recommended when estimating simple random samples. Participants were selected through a random sampling from a comprehensive list of women, provided by the Market Administrator. Each 6^th^ woman on the list was included in the study (K = 6 = number of women/sample size). Women in the second or third trimester of pregnancy were excluded from the study, as their BMIs cannot be compared with those of non-pregnant women. We have also excluded women who had tuberculosis, according to their own statements, or HIV/AIDS, according to 7 standard questions on clinical criteria defined by WHO that were asked during the interview, as these conditions also might influence BMI.

**Data collection**: Data were collected in October 2014 within the central market of Lubumbashi. Participants were interviewed individually for 20 to 30 minutes and the interview tool was adapted from the version 3.1_2014 of the WHO Stepwise approach surveillance instrument [20], which included questions on socio-demographic characteristics, smoking, alcohol intake, fruit and vegetable consumption, physical activity, current or previous use of birth control pills and self-reported previously diagnosed heart disease and diabetes. A quantitative 24-hours recall of fruit and vegetable consumption was used to compute the number of servings of fruits and vegetables consumed per week, as the sum of the average daily consumption of fruits and vegetables. A trained professional was appointed to measure weight, height and blood pressure of selected women and 4 trained interviewers were in charge of questionnaire administration. Women's heights and weights were measured with an electronic scale (SECA 1200, Seca Co. Hamburg, Germany), equipped with portable height measuring stick, on which women stood barefoot without headgear and with minimal clothing according to the FANTA (Food and Nutrition Technical Assistance) protocol [21]. Measured weight and height were used to calculate BMI (kg/m²). Participants were classified into 4 groups: underweight (BMI < 18.5), normal weight (BMI 18.5-24.9), overweight (BMI 25.0-29.9), or obese (BMI ≥ 30.0) [22]. Respondents were considered to be smokers if they reported currently smoking. We used the International Physical Activity questionnaire to classify respondents into 4 groups of physical activity: 1) vigorous, 2) moderate, 3) insufficient and 4) none [23]. Blood pressure was recorded with an electronic blood pressure measurer (BEURER GmbH, Gerätebau GmbH, Germany). Measurements were taken with the participant resting, at 5 minutes intervals. We followed the National Health and Nutrition Examination Survey procedures to edit and determine blood pressure levels [24]. Respondents were considered to be hypertensive if they met any of the following criteria: 1) measured systolic blood pressure exceeding 140 mmHg and/or diastolic blood pressure exceeding 90 mm Hg; or 2) measured diastolic or systolic blood pressure not exceeding the appropriate threshold but the respondent reports use of blood pressure medication.

**Data analysis**: Analysis was conducted with STATA 14. A multivariate logistic regression model was fitted, to compare socio-demographic characteristics and BMI categories in the population. The association between obesity and related factors was measured by using a backward elimination multivariate logistic regression model. All factors were included in the model and then eliminated, based on the Breusch-Pagan x^2^ test for analysis of independence [25]. Variables were removed one by one, based on the significance level of their effect on the model, starting by those with the highest p value > 0.05, until all remaining had a value of p < 0.05 in the analysis of independence. To avoid missing data, the survey supervisor crosschecked the questionnaire on a daily basis and if data were found missing, the interviewer was asked to return and complete the questionnaire and this explains the 100% response rate reported in this study.

**Ethics**: Ethical clearance was obtained from the medical ethic committee of the faculty of medicine at University of Lubumbashi, DRC. Informed consent was obtained during recruitment from each woman, by signature or thumb stamp in case the participant was unable to sign. Privacy and confidentiality were respected and none of personal information have been reported.

## Results

**Figure 1:**
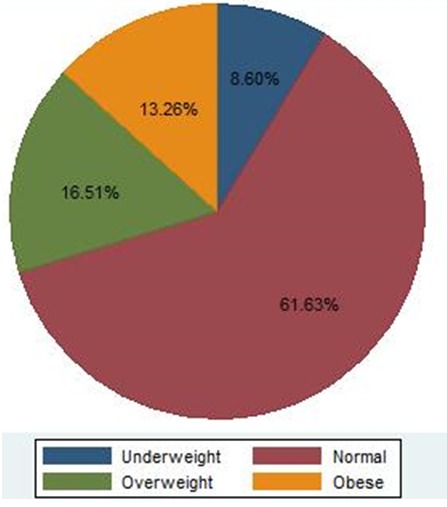
Distribution of BMI categories of respondents

In October 2014, we interviewed a total of 430 women selling in the central market of Lusonga in Lubumbashi. All 430 participants have completed the survey, given that women in the market were very keen to participate in the study (response rate 100%). Main characteristics of participants are presented in Table 1. Almost 52% of women in the sample were from the suburban area and 57.67% were married. Age of 65% of respondents was between 30 and 49 years; mean age was 40.7 years (SD: 11.10 years). Forty-four percent of respondents completed primary school and 47.91% attended secondary school. The prevalence of use of oral birth control pills was 15.35%. 3.72% of respondents reported smoking. Almost 2 respondents out of 5 reported alcohol consumption (39.07%) and sufficient fruits (40.47%) and vegetables (37.91%) intake. This study showed that 56.05% of participant had a low, or no physical activity profile (Table 1). The overall mean BMI was 23.9 kg/m^2^ ((95% CI: 23.5-24.4); SD: 5.0); prevalence of overweight and obesity was 16.51% and 13.26% respectively. Overweight and obesity together presented a prevalence of 30% (Figure 1).

**Table 1:** Characteristics of study participants

Variable	Frequency (n=430)	Percent
**Area**		
Suburban	223	51.86
Urban	207	48.14
**Age**		
20-29 years	64	14.88
30-39 years	154	35.82
40-49 years	127	29.53
50-59 years	56	13.03
≥60 years	29	6.74
Mean (SD)	40.7 (11.1)	Range: 20-72
**Marital status**		
Single	82	19.07
Married	248	57.67
Divorced	40	9.30
Widowed	60	13.95
**Educational level**		
None	8	1.86
Primary	189	43.95
Secondary	206	47.91
High	27	6.28
**Oral contraceptive**		
No	364	84.65
Yes	66	15.35
**Diabetes mellitus**		
No	354	82.33
Yes	76	17.67
**Heart disease**		
No	358	83.26
Yes	72	16.74
**Smoking**		
No	414	96.28
Yes	16	3.72
**Alcohol intake**		
No	262	60.93
Yes	168	39.07
**Vegetable intake**		
No	267	62.09
Yes	163	37.91
**Fruit intake**		
No	256	59.53
Yes	174	40.47
**Physical activity**		
None	175	40.70
Low	66	15.35
Moderate	141	32.79
High	48	11.16
**Systolic Blood Pressure**		
Normal	332	77.21
Elevated	98	22.79
Mean (SD)	124.0 (18.87)	Range: 81.0-169.0
**Diastolic Blood Pressure**		
Normal	327	76.05
Elevated	103	23.95
Mean (SD)	78.2 (13.6)	Range: 49.0-102.0
**Eleveted Blood Pressure**		
No	322	74.88
Yes	108	25.12

**Figure 2:**
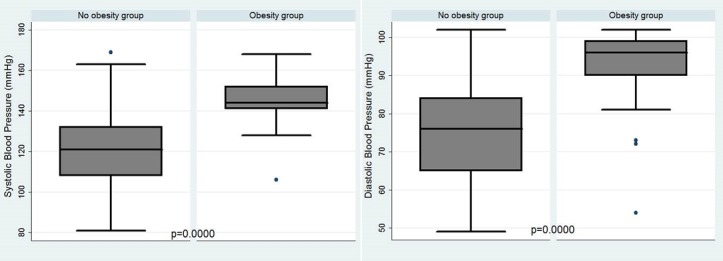
Distribution of systolic and diastolic blood pressure among obese and no obese women

**Risk factors for obesity**: Table 2 shows the relationship between obesity and various factors (sociodemographic, diet indicators and physical activity). In the logistic regression model, risk of obesity was lower among married women. Women who had a primary school or less educational profile were more likely to be obese than those who were more educated (secondary or high school) (adjusted odds ratio (aOR) = 2.50 (1.12-5.63)). Risk of obesity increased with living in urban area (aOR = 2.52 (1.00-6.36)), use of oral birth control pills (aOR = 11.07 (3.52-34.83)) and low fruit (aOR = 5.47 (1.88-15.92)) and vegetable intake (aOR = 2.42 (1.05-5.56)). Physical activity was not significantly associated with obesity in this study. Mean values of blood pressure were 124 mmHg ((95%CI: 122.22 - 125.79); SD: 18.87) for systolic blood pressure and 78.2 mmHg ((95%CI: 76.91 - 79.49); SD: 13.6) for diastolic blood pressure. The prevalence of hypertension was 25.12% (95%CI: 21.14 - 29.54%) (Figure 1). Obese women had mean systolic and diastolic blood pressure (145.50 mmHg (95%CI: 142.63 - 148.39); SD: 11.25 mmHg and 93.26 (95%CI: 90.97 - 95.55); SD: 8.95 mmHg respectively) that were significantly higher compared to non-obese (120.45 (95%IC: 118.66- 122.24); SD: 17.47 mmHg and 75.71 (95%CI: 74.42-77.0); SD: 12.59 mmHg respectively) (p < 0.0001) (Figure 2). Prevalence of diabetes and cardiovascular disease were 17.67% and 16.74% respectively. Obese women were more likely to be hypertensive (aOR = 7.15 (2.46-20.75)) and diabetics (aOR = 3.62 (1.62-8.11)) than non-obese.

**Table 2:** Predictors of obesity

Variable		Obesity (n=57)	No obesity (n=373)	Total (N=430)	Crude OR [CI95%]	Adjusted OR [CI95%]
Marital status	Single	29 (15.93)	153 (84.07)	182	1.00	1.00
	Married	28 (11.29)	220 (88.71)	248	0.67 [0.38-1.17]	0.23 [0.08-0.63]
Age	<40 years	15 (6.88)	203 (93.12)	218	1.00	1.00
	≥40 years	42 (19.81)	170 (80.19)	212	3.34 [1.79-6.23]	1.65 [0.59-4.55]
	Mean (SD)	48.11 (10.11)	39.63 (10.82)	40.70 (11.09)		
Area	Urban	47 (22.71)	160 (77.29)	207	6.25 [3.07-12.76]	2.52 [1.00-6.36]
	Suburban	10 (4.48)	213 (95.52)	223	1.00	1.00
Educational level	No educate/Primary	37 (15.88)	196 (84.12)	233	1.67 [0.93-2.98]	2.50 [1.12-5.63]
	Secondary/High	20 (10.15)	177 (89.85)	197	1.00	1.00
Oral contraceptive	Yes	30 (45.45)	36 (54.55)	66	10.40 [5.57-19.40]	11.07 [3.52-34.83]
	No	27 (7.42)	337 (92.58)	364	1.00	1.00
Diabetes mellitus	Yes	32 (42.11)	44 (57.89)	76	9.57 [5.19-17.62]	3.62 [1.62-8.11]
	No	25 (7.06)	329 (92.94)	354	1.00	1.00
Eleveted Blood Pressure	Yes	46 (42.59)	62 (57.41)	108	20.97 [10.29-24.75]	7.15 [2.46-20.75]
	No	11 (3.42)	311 (96.58)	322	1.00	1.00
Heart disease	Yes	27 (37.50)	45 (62.50)	72	6.56 [3.58-12.02]	1.06 [0.46-2.47]
	No	30 (8.38)	328 (91.62)	358	1.00	1.00
Smoking	Yes	2 (12.50)	14 (87.50)	16	0.93 [0.20-4.21]	1.11 [0.18-6.80]
	No	55 (13.29)	359 (86.71)	414	1.00	1.00
Alcohol intake	Yes	39 (23.21)	129 (76.79)	168	4.09 [2.25-7.45]	1.97 [0.89-4.33]
	No	18 (6.87)	244 (93.13)	262	1.00	1.00
Physical activity	Low	54 (22.41)	187 (77.59)	241	17.90 [5.50-58.28]	4.04 [0.88-18.40]
	High	3 (1.59)	186 (98.41)	189	1.00	1.00
Fruit intake	Low	50 (28.74)	124 (71.26)	174	14.34 [6.31-32.56]	5.47 [1.88-15.92]
	High	7 (2.73)	249 (97.27)	256	1.00	1.00
Vegetable intake	Low	41 (25.15)	122 (74.85)	163	5.27 [2.84-9.77]	2.42 [1.05-5.56]
	High	16 (5.99)	251 (94.01)	267	1.00	1.00

## Discussion

The prevalence of obesity is rapidly increasing in developing countries and according to WHO, this condition would have affected more than 600 million people in these countries in 2014 [26]. Women are more affected than men because of their anatomy, physiology and metabolism; which predispose them to obesity, in addition to the fact that in Africa they often have a sedentary lifestyle [27]. The aim of this study was to determine the prevalence of obesity and its risk factors, in women selling in Lusonga market in Lubumbashi, DRC. The prevalence of obesity was 13.26%; which is much lower than those reported for women in Arabic countries ranging from 30.6 to 49% [28-31]. In 2010, a study in urban area of Cameroon showed a prevalence of 33% of obesity among adult women [32]. Although the prevalence of obesity reported in this study is lower than that found by other authors elsewhere, it is however the double of the prevalence of 7.1% reported by WHO in 2014, for adult women in the general population of the DRC [33]. According to Rahim, the high prevalence found in his study could mainly result from sociocultural factors which gives a positive connotation to overweight in the south of Morocco [30]. Same holds true for Nono, who found that weight gain seems to be an indicator of well-being among married women, particularly according to local beliefs in Limbe (Cameroon) [32]. The desire for physical appearance in women is not a personal choice, but a deep feeling that is subject to socio-cultural pressure and changes from one culture to another. It is through the eyes of others that overweight and obesity become socially acceptable [30]. In sub-Saharan Africa, the belief that weight loss is associated with Acquired Immune Deficiency Syndrome (AIDS) is widespread [34]. Puoane said that South African women are not motivated to engage in physical activity because they fear weight loss and being stigmatized as having AIDS [35]. The notion that obesity and overweight are seen as a sign of good health is not so surprising, especially in countries where malnutrition and infectious diseases such as HIV are endemic [34]. This study showed that a number of factors are associated with obesity in women of the Lusonga market in Lubumbashi. The multivariate analysis revealed that marital status, education, residence, use of oral birth control pills, fruit and vegetables intake, had a significant association with the prevalence of obesity in this population.

Associations found in our study are unlike to findings from previous studies, with varying results between women. The association between marital status and obesity has been previously cited. Despite being generally in better health than their unmarried counterparts, married women were found to be at higher risks for obesity [29]. In this study, never married women, as well as divorced, or widowed, were more likely to be obese than married women. Results of our study show that marriage was a protective factor against obesity. Women living alone (single, widowed or divorced) were more likely to become obese than married. This finding is contrary to previous studies [29, 36, 37]. In the Moroccan study women declared that a fat and fleshy woman is the ideal type of beauty for men and a condition for their marriage and meant to be healthy and rich [30]. We believe that oral birth control pills (frequently used by unmarried women [38]), could explain the significant association noted between obesity and living alone in our series. Our results show that women's educational level had a significant positive association with obesity: a low level of education means a significantly increased likelihood for obesity. This is also correlated with the findings of earlier studies that assessed how obesity varies by level of education, the results of which suggest that low-educated women are more likely to be obese than women with higher educational profile [29, 30, 39]. Memish noted that, with higher education, Saudi women can make healthier choices that reflect their body composition and added that more educated women tend to improve their health profiles and that of their children by adopting a healthy living mode [29]. On the contrary, other studies have shown that women´s low educational attainment was negatively correlated with obesity [40, 41]. Women living in urban areas were at higher risk of being obese than those living in rural area. Urbanization has been significantly associated with obesity. This finding is consistent with other studies [42, 43]. Urbanization in developing countries has been associated with lifestyle changes that lead to increased consumption of high-calorie foods and has resulted in several environmental factors that generate a less active lifestyle that can be summarized as an increase in sedentary behaviors during professional activities, decreased physical activity during leisure time and increased use of passive means of transportation [34].

As in the study by Park and Kim [38], our study reports that the use of oral contraception has been found to be a significant predictor of obesity among women in our series. Rosenberg believes that the increase in weight associated with oral contraception may be related to the estrogenic stimulation of the renin-angiotensin mechanism and the retention of fluids in the body [44]. In addition, there may be an increase in subcutaneous fat, especially in breast, hips and thighs in estrogen users and an increased appetite (due to anabolic properties) in those using Progestin [45]. As in the Memish's study, our study also found a significant association between obesity and diabetes mellitus [29]. Obesity is a well-known risk factor for the development of type 2 diabetes. Metabolically, it is most often associated with insulin resistance, a stage preceding the onset of type 2 diabetes. This insulin resistance is linked to the infiltration of tissues, in particular muscles by lipids, caused by a permanent and increased flow of free fatty acids. These metabolic disturbances are generally accompanied by mitochondrial dysfunction of the skeletal muscle, that has recently been considered as the main causative factor for metabolic pathologies associated with obesity [46]. Our results corroborate those reported elsewhere, which show that increased daily intake of fruits and vegetables is often associated with decreased risk of obesity [47]. The “anti-obesity” character of fruits and vegetables is gradually asserting itself, despite the fact that its mechanism is still vague. A joint WHO/FAO expert report indicates that fruits and vegetables reduce the risk of obesity and that they are likely to reduce the risk of diabetes. These experts recommend a minimum intake of 400 g of fruit and vegetables per day for the prevention of chronic diseases such as heart disease, cancer, diabetes and obesity [48, 49]. Our study has some limitations. First, data are cross-sectional, so we have not been able to ascertain causality in associations. Secondarily, chronic conditions were self-reported, which leaves out respondents who might have these conditions but have not been previously diagnosed. Thirdly, many of our behavioral data, such as diet and physical activity, are self-reported and subject to recall and social desirability biases. Conversely, our study is based on a large sample size and used standardized methods for all its measures.

## Conclusion

Obesity is a risk factor for morbidity and mortality. This study has reported a prevalence of 13.26% of obesity among women selling at the central market of Lusonga in Lubumbashi, Democratic Republic of Congo. Findings in this study call for efforts to mitigate the prevalence of obesity and its risk factors in this category of population, by targeting identified high-risk individuals such as unmarried women, those with low educational profile, those living in urban areas, those using oral contraception and those with low fruits and vegetables intake. Results from this study also raises a need to determine how and why physical activity was not significantly associated with obesity in this population.

### What is known about this topic

- Obesity is known as one of adjuvant factors for increase in NCDs;
- In many developing societies, the adoption of a Western lifestyle, with decreased physical activity and high caloric intake, is contributing to an alarming epidemiological transition marked by the shift in the leading causes of death from communicable to NCDs.

### What this study adds

- Our study is the first to describe prevalence of obesity and to identify its risk factors among women in Lubumbashi, Democratic Republic of Congo;
- Findings in this study call for efforts to mitigate the prevalence of obesity, its risk factors among this category of women, by targeting identified high-risk individuals such as are unmarried women, those with low level of education, those living in urban areas, those using oral contraception and those with low fruits and vegetables consumption.

## Competing interests

The authors declare no competing interest.
